# A novel effective method for the assessment of microvascular function in male patients with coronary artery disease: a pilot study using laser speckle contrast imaging

**DOI:** 10.1590/1414-431X20165541

**Published:** 2016-09-01

**Authors:** J.P. Borges, G.O. Lopes, V. Verri, M.P. Coelho, P.M.C. Nascimento, D.A. Kopiler, E. Tibirica

**Affiliations:** 1 Laboratório de Atividade Física e Promoção è Saúde, Departamento de Desporto Coletivo, Instituto de Educação Física e Desportos, Universidade do Estado do Rio de Janeiro, Rio de Janeiro, RJ, Brasil; 2 Instituto Nacional de Cardiologia, Rio de Janeiro, RJ, Brasil; 3 Laboratório de Investigação Cardiovascular, Departamento Osório de Almeida, Instituto Oswaldo Cruz, FIOCRUZ, Rio de Janeiro, RJ, Brasil

**Keywords:** Ischemic heart disease, Cutaneous vascular conductance, Microvascular flowmetry, Endothelial function, Post-occlusive reactive hyperemia

## Abstract

Evaluation of microvascular endothelial function is essential for investigating the pathophysiology and treatment of cardiovascular and metabolic diseases. Although laser speckle contrast imaging technology is well accepted as a noninvasive methodology for assessing microvascular endothelial function, it has never been used to compare male patients with coronary artery disease with male age-matched healthy controls. Thus, the aim of this study was to determine whether laser speckle contrast imaging could be used to detect differences in the systemic microvascular functions of patients with established cardiovascular disease (n=61) and healthy age-matched subjects (n=24). Cutaneous blood flow was assessed in the skin of the forearm using laser speckle contrast imaging coupled with the transdermal iontophoretic delivery of acetylcholine and post-occlusive reactive hyperemia. The maximum increase in skin blood flow induced by acetylcholine was significantly reduced in the cardiovascular disease patients compared with the control subjects (74 *vs* 116%; P<0.01). With regard to post-occlusive reactive hyperemia-induced vasodilation, the patients also presented reduced responses compared to the controls (0.42±0.15 *vs* 0.50±0.13 APU/mmHg; P=0.04). In conclusion, laser speckle contrast imaging can identify endothelial and microvascular dysfunctions in male individuals with cardiovascular disease. Thus, this technology appears to be an efficient non-invasive technique for evaluating systemic microvascular and endothelial functions, which could be valuable as a peripheral marker of atherothrombotic diseases in men.

## Introduction

Cardiovascular diseases are among the leading causes of morbidity and mortality worldwide (1). Atherosclerosis is a major disease that can lead to ischemia of the heart, brain or extremities, resulting in organ damage or infarction. The pathophysiology of atherosclerosis comprises a series of highly specific cellular and molecular responses that can be defined as an inflammatory disease and may be present throughout a person's lifetime (2). Endothelial dysfunction precedes clinically detectable atherosclerosis and can also contribute to arterial lesion development and later clinical complications (3). Therefore, the evaluation of microvascular endothelial function is essential for investigating the pathophysiology of cardiometabolic diseases, including arterial hypertension, diabetes, dyslipidemia and obesity (4).

Nonetheless, from a clinical perspective, the development of an easy-to-perform and noninvasive test for routinely assessing microvascular endothelial function is still required. An optimal tool for routine use should, among other factors, be non-invasive, specific, and able to detect diseased patients (3). In this sense, the more common non-invasive methods that have been developed in clinical microvascular laboratories are based on laser technology, such as laser Doppler imaging and laser Doppler flowmetry (4–6). However, measuring microvasculature function presents a particular challenge because the vessel structure is spatially inhomogeneous, and perfusion can be highly variable over time (6). Previous studies have suggested that these features are related to the high variability of measurements based on single-point methods, including laser Doppler technology measurements (5,7,8).

Recently, another non-invasive method for measuring microvascular blood flow termed laser speckle contrast imaging (LSCI) has gained increasing attention. This methodology has previously been used to evaluate blood flow in experimental settings (9–11) and in the human brain (12,13). The LSCI method was first introduced in the 1980s and is a powerful tool for the full-field imaging of blood flow (14). In contrast to laser Doppler technologies, this method consists of assessing the blood flow response over a broad area of analysis rather than at a single point (5). This broad area of analysis is particularly important because it reduces the variability of the measurements due to the spatial heterogeneity of the skin microvasculature, especially in response to drug delivery (5). Indeed, previous studies have shown that the reproducibility of LSCI coupled with post-occlusive reactive hyperemia (PORH) (5) and the iontophoresis of acetylcholine are excellent (3).

Many studies have examined the methodological issues of LSCI (15–17) and compared different methods in terms of their abilities to assess blood flow in healthy individuals (3,18,19). Nonetheless, previous data regarding the ability of LSCI to detect diseased patients are limited (4,20). Thus, our aim was to determine whether LSCI is an efficient method for identifying impairments in skin microvascular function in male patients with coronary artery disease (CAD) compared to age-matched healthy individuals.

## Material and Methods

### Ethics

All procedures described in the present study were conducted in accordance with the Declaration of Helsinki of 1975 as revised in 2000 and were approved by the Institutional Review Board (IRB) of the Instituto Nacional de Cardiologia do Rio de Janeiro, Brazil (protocol #894.911). Once considered eligible, all subjects signed an informed consent form that was approved by the IRB.

### Subjects

A sample of 61 consecutive male patients with CAD (58.9±12.8 years old) from the Cardiac Rehabilitation Program of the Instituto Nacional de Cardiologia, Rio de Janeiro, Brazil and 24 healthy male individuals (56.1±4.9 years old), randomly recruited from the staff of the same institution, were enrolled in this cross-sectional study.

In the CAD patients, 67% had previous acute myocardial infarction and referred diagnoses of diabetes (44%), arterial hypertension (80%), and dyslipidemia (65%) according to their medical records. In the control subjects, all of the abovementioned pathologies were absent. CAD was defined by the occurrence of any acute coronary syndrome, including ST and non-ST elevation myocardial infarction, and unstable angina (all defined by characteristic histories and electrocardiographic and cardiac enzyme abnormalities) or by the diagnosis of obstructive CAD based on coronary angiography (defined as ≥50% stenosis of any epicardial coronary artery) in patients with stable angina.

According to the study protocol, the patients and controls underwent cutaneous microvascular reactivity testing. On the morning scheduled for the test, the patients presented in a 12-h fasted condition for blood collection. The patients must not have smoked or ingested caffeine from the night before until the completion of the tests. The patients took their usual medications on the morning of the tests, except direct vasodilators, which were administered immediately after the microcirculatory tests.

### Evaluation of skin microvascular reactivity using LSCI

**Figure 1:**
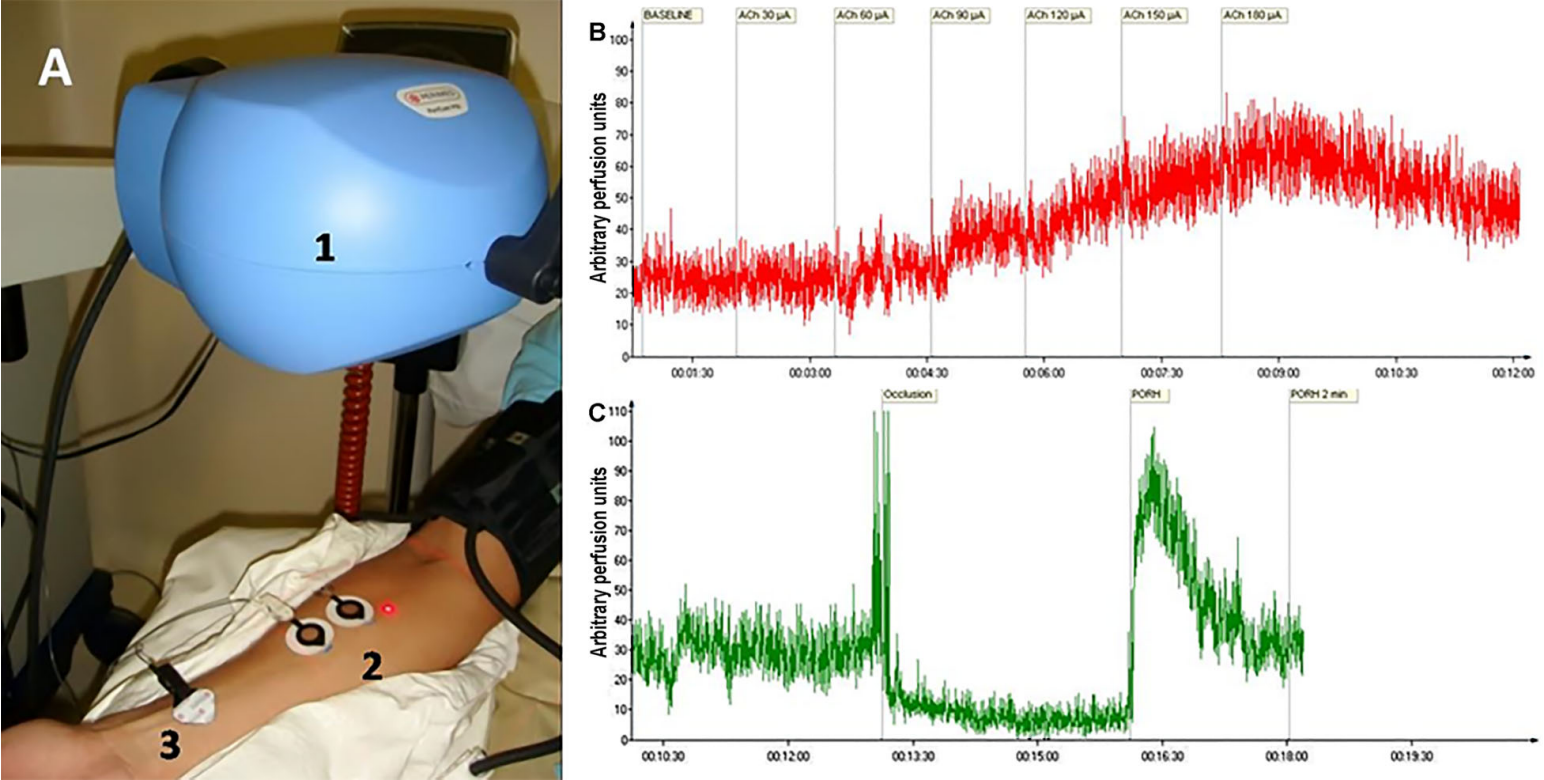
*A*, Photograph of the experimental set up used in the assessment of skin microvascular perfusion using laser speckle contrast imaging coupled with iontophoresis of vasodilator drugs. 1) Imager head, 2) drug-delivery iontophoresis electrodes, 3) dispersive electrode (see Material and Methods). *B*, Representative examples of the effects of the transdermal iontophoretic delivery of cumulative doses of acetylcholine (ACh) on skin blood flow during the iontophoresis of ACh (2% w/v) using increasing anodal currents of 30, 60, 90, 120, 150, and 180 μA applied in 10-s intervals spaced 1 min apart. *C*, skin microvascular blood flow during post-occlusive reactive hyperemia (PORH).

Microcirculatory tests were performed after a 20-min rest with the patients in the supine position in a temperature-controlled room (23±1°C) approximately 1 h after a light breakfast. Microvascular reactivity was evaluated using a LSCI system with a laser wavelength of 785 nm (PeriCam PSI system, Perimed, Sweden) in combination with the iontophoresis of acetylcholine (ACh) for noninvasive and continuous measurements of cutaneous microvascular perfusion changes (in arbitrary perfusion units, APU; Figure 1) (4,20). The image acquisition rate was 8 images/s, and the distance between the laser head and the skin surface was fixed at 20 cm as recommended in the manufacturer's manual. Images were analyzed using the manufacturer's software (PIMSoft, Perimed).

The skin sites used for the microvascular flow recordings were randomly chosen on the ventral surface of the forearm, and hair, broken skin, areas of skin pigmentation and visible veins were avoided. The drug-delivery electrode was secured using an adhesive disc (LI 611, Perimed). Two measurement areas (circular regions of interest) of approximately 80 mm^2^ were examined. One of the measurement areas was within the electrode area (for the ACh-related measurements), and the other was adjacent to the electrode (for the PORH measurements). A vacuum cushion (a specially constructed pillow filled with polyurethane foam that can be molded to any desired shape by creating a vacuum; from AB Germa, Sweden) was used to reduce recording artifacts generated by arm movements. ACh (2% w/v; Sigma Chemical Co., USA) iontophoresis was performed using a micropharmacology system (PF 751 PeriIont USB Power Supply, Perimed) with increasing anodal currents of 30, 60, 90, 120, 150, and 180 μA applied in 10-s intervals spaced 1 min apart (the total charges were 0.3, 0.6, 0.9, 1.2, 1.5, and 1.8 mC, respectively). The dispersive electrode was attached approximately 15 cm away from the electrophoresis chamber. Of note, the drug was not injected but rather was placed in contact with the skin surface.

During the PORH test, arterial occlusion was performed with suprasystolic pressure (50 mmHg above the systolic arterial pressure) using a sphygmomanometer over 3 min. Following the release of the pressure, the maximum flux was measured. The measurements of skin blood flow were divided by the mean arterial pressure to yield the cutaneous vascular conductance (CVC) in APU/mmHg. The amplitudes of the PORH responses were expressed as the peak CVC minus the baseline CVC.

### Statistical analysis

The results are reported as means±SD. Normal sample distributions were confirmed with the Shapiro-Wilk test. Comparisons between groups were performed using the two-tailed unpaired Student's t-test. The null hypothesis was rejected at P<0.05. The Prism version 5.0 statistical package (GraphPad Software Inc., USA) was employed.

## Results

### Subject characteristics

Baseline clinical characteristics are presented in Table 1. The healthy volunteers exhibited higher values of the total, LDL and HDL cholesterol and lower glucose values compared with the CAD patients. Concerning the use of cardiovascular drugs, 28% of the CAD patients used angiotensin-converting enzyme inhibitors, 90% used β-blockers, 92% used lipid-lowering drugs, 36% used nitrates, 52% used angiotensin II receptor blockers, and 57% used diuretics.

**Table:**
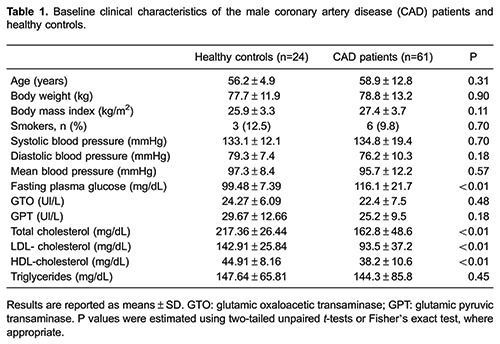

### Skin microvascular reactivity

**Figure 2:**
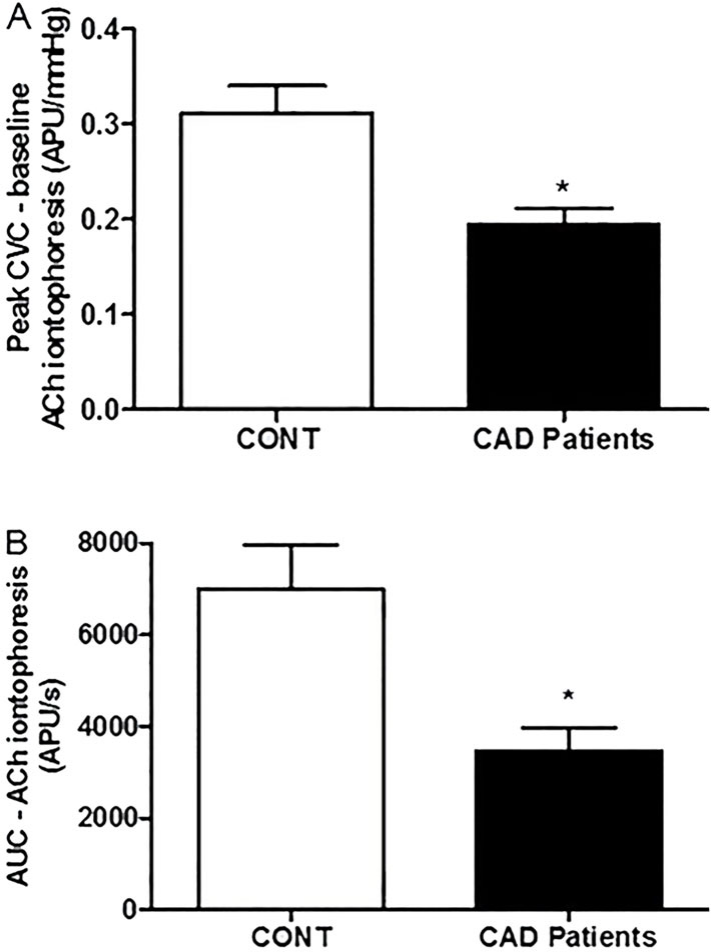
Effects of the skin iontophoresis of acetylcholine (ACh) on cutaneous microvascular conductance [CVC, expressed in arbitrary perfusion units (APU) divided by mean arterial pressure in mmHg] in healthy volunteers (CONT) and male patients with coronary artery disease (CAD). *A*, peak CVC minus baseline CVC resulting from the iontophoresis of ACh. *B*, area under the curve (AUC) for the iontophoresis of ACh on the skin. Data are reported as means±SE. *P<0.05, compared to control (two-tailed unpaired Student's *t*-test).

The mean resting CVC did not differ between the groups (healthy subjects: 0.27±0.06 *vs* CAD patients: 0.29±0.08 APU/mmHg; P=0.21). The patients' microvascular responses to ACh were significantly reduced compared with those of the healthy subjects; the maximum increase in CVC induced by ACh in the patients was 74% compared with 116% in the controls (P=0.002). The increases in CVC relative to baseline during the iontophoresis of ACh were 0.31±0.14 and 0.19±0.14 APU/mmHg in the control and CAD participants, respectively (P=0.001; Figure 2A). The areas under the curves for the ACh-induced vasodilation were 7005±4493 and 3469±3075 APU/s in the control and CAD participants, respectively (P=0.001; Figure 2B).

**Figure 3:**
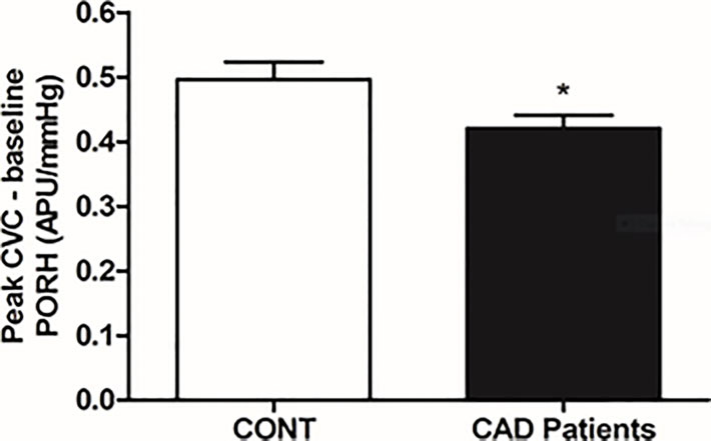
Effects of post-occlusive reactive hyperemia (PORH) on skin cutaneous microvascular conductance [CVC, expressed in arbitrary perfusion units (APU) divided by mean arterial pressure in mmHg] in healthy volunteers (CONT) and male patients with coronary artery disease (CAD). *P<0.05, compared to control (two-tailed unpaired Student's *t*-test).

Concerning the microvascular reactivity to PORH, the CAD patients also presented lower responses than the healthy participants (0.42±0.15 and 0.50±0.13 APU/mmHg, respectively; P=0.04; Figure 3).

## Discussion

The main finding of this study is that LSCI is capable of identifying the reduced endothelium-dependent skin microvascular vasodilator responses in patients with CAD compared with healthy subjects.

Our results are relevant to medical research because the evaluation of microvascular endothelial function is essential for investigating the pathophysiology of cardiometabolic diseases, including arterial hypertension and diabetes (4). In this context, LSCI is a recently developed technique that is based on laser speckle contrast analysis (LASCA) and provides an index of microvascular blood flow (7). LASCA is based on the principle that when an object is illuminated by a coherent light, such as laser, the light will be scattered by a collection of randomly distributed particles to produce a characteristic random interference pattern known as a speckle pattern that consists of light and dark areas (21). If the illuminated object is static, the speckle pattern is stationary. When there is object movement, such as red blood cells in a tissue, the speckle pattern will change overtime. The level of blurring will differ according to the degree of movement; greater movement elicits greater blurring of the speckle pattern. The level of blurring is quantified by the speckle contrast. By acquiring an image of the speckle pattern and quantifying the blurring of the speckles via measurements of the spatial intensity variations in the speckle pattern, spatial maps of the relative blood flow can be obtained (21,22).

Because of its high level of reproducibility and advantages of good spatial and temporal resolutions, LSCI plays a key role in the analysis of microvascular function, especially when coupled with PORH and non-invasive transdermal drug delivery (iontophoresis) (6,7,23). It is also noteworthy that the cutaneous microcirculation is now considered to be an accessible and representative vascular bed for assessments of systemic microcirculatory reactivity (8,24,25). Moreover, alterations of microvascular function in the skin have previously been demonstrated to correlate with increased CAD risk (26).

Many investigations have focused on verifying the day-to-day repeatability and inter-subject reproducibility of LSCI and comparing different methodologies in terms of the interpretations of the experimental results (3,5,11,18,19,27). Nevertheless, only two studies have compared the systemic microvascular endothelial function of patients with that of healthy individuals using LSCI (4,20). The study by Cordovil et al. (4) was the first to validate this methodology for the evaluation of cardiovascular and metabolic diseases. These authors first suggested that LSCI could be a useful noninvasive technique for evaluating systemic microvascular endothelial function. These authors demonstrated that patients with arterial hypertension and severe dyslipidemia exhibit a reduction of 47% in the maximum increase in systemic microvascular blood flow induced by ACh compared with controls (4). In the present study, this response in CAD patients was reduced by 36% compared with the control subjects. The difference in the results between studies is probably attributable to the fact that the previous study did not compare patients with an age-matched control group. In addition to cardiovascular diseases, age differences between groups undoubtedly contributed to the observed endothelial dysfunction. Indeed, aging is well known to be associated with a progressive deterioration in endothelial function (28). Thus, a major strength of the current study compared with previous research is that LSCI was used for the first time to compare male patients with established CAD and healthy age-matched subjects. Although LSCI technology had been previously validated (4), we demonstrated that LSCI is still an efficient tool for the detection of endothelial dysfunction even when patients with compromised microvascular endothelial function are compared with healthy counterparts of the same gender and age.

A previous study from our group compared the skin microvascular functions in individuals with early-onset CAD and healthy individuals using LSCI (20). Our results agree with the results of Souza et al. (20) and demonstrate that the endothelium-dependent skin microvascular vasodilator responses induced by both ACh and PORH are significantly reduced in patients with premature CAD compared with age-matched healthy individuals. These latter results suggest that LSCI could become a useful non-invasive technology for the development of early markers of microvascular endothelial dysfunction in cardiovascular disease.

The absence of women in our sample is the major limitation of the present study. Cutaneous blood flow may vary according to the hormonal oscillations of the menstrual cycle and therefore interfere with LSCI data. This is important because the present results should be interpreted with caution when evaluating women, especially those who did not achieve menopause age.

In conclusion, our data indicated that LSCI was capable of identifying endothelial microvascular dysfunctions in male individuals who present cardiovascular diseases. Thus, LSCI appears to be an efficient non-invasive technique for evaluating systemic microvascular and endothelial functions, which could be valuable as a peripheral marker of atherothrombotic diseases in men. Further research investigating its use in women with cardiovascular diseases is certainly warranted.
